# A Simultaneous Modulation of Reactive and Proactive Inhibition Processes by Anodal tDCS on the Right Inferior Frontal Cortex

**DOI:** 10.1371/journal.pone.0113537

**Published:** 2014-11-26

**Authors:** Toni Cunillera, Lluís Fuentemilla, Debora Brignani, David Cucurell, Carlo Miniussi

**Affiliations:** 1 Department of Basic Psychology, University of Barcelona, Barcelona, Spain; 2 Cognition and Brain Plasticity Unit, Institute of Biomedicine Research of Bellvitge (IDIBELL), L'Hospitalet de Llobregat, Spain; 3 Cognitive Neuroscience Section, IRCCS Centro San Giovanni di Dio Fatebenefratelli, Brescia, Italy; 4 Neuroscience Section, Department of Clinical and Experimental Sciences, University of Brescia, Brescia, Italy; University of Bologna, Italy

## Abstract

Proactive and reactive inhibitory processes are a fundamental part of executive functions, allowing a person to stop inappropriate responses when necessary and to adjust performance in in a long term in accordance to the goals of a task. In the current study, we manipulate, in a single task, both reactive and proactive inhibition mechanisms, and we investigate the within-subjects effect of increasing, by means of anodal transcranial direct current stimulation (tDCS), the involvement of the right inferior frontal cortex (rIFC). Our results show a simultaneous enhancement of these two cognitive mechanisms when modulating the neural activity of rIFC. Thus, the application of anodal tDCS increased reaction times on Go trials, indicating a possible increase in proactive inhibition. Concurrently, the stop-signal reaction time, as a covert index of the inhibitory process, was reduced, demonstrating an improvement in reactive inhibition. In summary, the current pattern of results validates the engagement of the rIFC in these two forms of inhibitory processes, proactive and reactive inhibition and it provides evidence that both processes can operate concurrently in the brain.

## Introduction

A key aspect of executive functions is behavioral inhibition, the ability to control inappropriate or unwanted responses [1]. This ability constitutes the basis of accurate performance [2] and it is essential for adaptive behavior in everyday life. Indeed, there are many situations requiring to stop a specific response or to move in a controlled fashion. For instance, we can eat slowly in an etiquette dinner, even though being “famished”, and we can even react and stop eating if somebody asks us to do it or if we perceive a strange taste or smell in the food. While healthy adults are generally adept at inhibiting a wide-range of behaviors if needed, diminished capacity has been highlighted in aging [3] and clinical [4] populations. A better understanding of the underlying cognitive processes and its neural substrates would be the key to furthering the knowledge of this field and developing clinical treatments.

At the cognitive level, the stopping performance has been described as a mechanism in relation with the appearance of a signal –reactive inhibition–, and with the preparation to stop an upcoming response tendency in accordance with the goals of a person –proactive inhibition– [4]. These Reactive and Proactive inhibitory function can also be understood as global and selective processes, respectively, thereby not functioning in opponent but in a complementary mode [5], [6] and permitting a more suitable control on behavior relying on the necessities and instructions of the task. In fact, reactive and proactive processes are not considered to compete against each other and have been described as the two principal mechanisms of cognitive control in the Dual Mechanism Control (DMC) framework [7].

At the neural level, the inhibitory function in the brain is proposed to be implemented by a specific fronto-basal-ganglia circuit [11], [12]. Reactive inhibition has been associated with the activation of the subthalamic nucleus (STN) of the basal ganglia blocking its outputs with a widespread effect on the motor system [8], [9], which is reflected by a rapid and global suppression of behavior. The proactive control process has been associated with sustained activation in the prefrontal cortex (PFC), reflecting the active maintenance of task goals and a top-down bias to facilitate the processing of upcoming cognitive demanding events [10]. Functional neuroimaging studies have shown a large coincidence in the activation of the right inferior frontal cortex (rIFC), the presupplementary motor area (preSMA), and the STN of basal ganglia [9], with a clear overlapped network for tasks involving the recruitment of reactive or proactive inhibitory processes [10]. Among these cortical areas, the rIFC is considered to be a critical one for stopping and for the attentional control of behavior [8], [13], [14]. In fact, recent studies provided evidence of the importance of the rIFC for stopping behavior in the reactive inhibition using either transcranial magnetic stimulation (TMS) [15], [16] or transcranial direct current stimulation (tDCS) [17], [18]. Moreover, recent studies have shown that the role of rIFC can be parceled out, with appropriate experimental designs, into distinct cognitive functions [19], [20]. Nevertheless, the simultaneous contribution of the rIFC to reactive and proactive inhibitory control has not been proved yet, although assuming a general updating function for the rIFC [20], it has been postulated that the inhibitory function occurs along with other processes which are critical for updating behavior.

At the experimental level, response inhibition is often assessed using the Stop-Signal task (SST) or the Go/Nogo task (GNG). In the SST, subjects are instructed to stop responding immediately after the appearance of a specific signal, and its underlying inhibitory process is reactive inhibition. The effectiveness of a correct performance in the SST is understood as an independent [21] or an interactive [22] race between the “go process” and the “stop process” triggered by presentation of a go stimulus and a stop-signal, respectively. In a variant of the SST, in which is introduced a key informing the subject about the possible appearance of the stop signal in that trial [23], [24], or in tasks where the proportion of the stop signal overwhelms the proportion of go-trials in an experimental block [25], a proactive inhibition is recruited [4], slowing down reaction times (RTs) but usually increasing accuracy in the task. The time measurement that is assumed to reflect the effectiveness of response inhibition required for the stop-process to occur is estimated for individual subjects in the context of the race model and is called the Stop-Signal RT (SSRT). In contrast, in the GNG it is required the engagement of a distinct process triggered by a ‘NoGo’ signal. During the task subjects need to occasionally refrain from responding in front of certain stimuli, thus engaging in an internally driven (rather than externally driven, as in the SST) inhibitory process. In has been stated that proactive response-strategy adjustments are made just before a trial or before a series of trials [26], [27], and so, a GNG task demands the maintenance of a long lasting goal, which is most likely to correspond to a proactive control process. Thus, people may increase responses threshold or suppress motor output in situations in which stop signal is expected to occur in comparison with situations in which the stop signal is not expected to appear [23], [27]. Thus, it is hypothesized that subjects may balance the go and stop processes by adjusting the response threshold on go trials and this adaptation on the response threshold is reflected by increases in both go RT and accuracy [28]. While reactive inhibition is understood as a process that usually interferes with an already initiated response, proactive inhibition permits the subject to increment executive control over selective signals over a longer time frame. Thus, to accurately perform in distinct situations and experimental task, different cognitive operations may be engaged, which ultimately stem for the notion that inhibitory behavior may not be a unitary modus operandi.

In fact, several authors have challenged the idea of response inhibition as a “unitary” and isolated function in the last decade (see [29], for a review). For instance, an interesting new theoretical framework for understanding response inhibition states that non-inhibitory processes have a critical role in stopping responses [30]. Hence, the importance of monitoring for environmental relevant and irrelevant information for stop signals points out the significance of signal detection, and more specifically, the output of the sensory detection process for stopping behavior [30]. It is obvious that for the stop process to occur –either triggered externally or internally–, first the stop signal has to be identified. Thus, an interesting question is whether the complexity of the stimuli –e.g., discriminability– may affect the inhibitory response. In the current study, participants were engaged in a mixed SST and GNG task and in addition they needed to classify the command signal along two dimensions, with one involving a more difficult time-consuming discrimination than the other [31]. The outcome of the hard discrimination provided the information of whether or not to respond (Go-Nogo), and the outcome of the easy discrimination informed the subject about which hand to use if a response was called for. Osman and colleagues [31] demonstrated that both stimulus attributes were processed in parallel and that the Nogo response was delayed in the condition in which the stimuli were harder to discriminate. It is unclear, however, whether both reactive and proactive inhibitory processes will be affected in a similar way by the attributes of the stimuli, i.e., by the underlying signal detection and monitoring processes, but by manipulating the difficulty of perceiving the stimuli needed for the inhibitory function to proceed, we pursued to further characterize the so far postulated different inhibitory functions.

To examine the specific role of the rIFC in behavioral inhibition and the nature of the underlying cognitive mechanism and its related processes –either reactive or proactive inhibitory processes, or both, and how they are influenced by the signal detection and monitoring processes– we designed a new within-subject experimental task in which both externally and internally driven inhibitory processes were manipulated equivalently (via the combination of a SST and a GNG task) together with the manipulation of the difficulty in discriminating the stimuli and implemented anodal tDCS to the rIFC. We hypothesize that if both inhibitory processes are governed by rIFC, an increase in both reactive (reduction of the SSRT) and proactive inhibitory processes (increase of RT and/or reduction of commission and omissions) should be achieved, providing direct evidence that these two processes can operate simultaneously. Moreover, by contrasting how signal detection process affects the different outlined measurements on the inhibitory function, we expect to provide new data on the interrelation of response inhibition with other aspects of the executive function.

## Materials and Methods

### Participants

Twenty-two healthy volunteers (4 males; mean age = 21.2 years; S.D. = 2.7) participated in the experiment. All participants were right handed according to the Edinburgh handedness inventory test [32] and had normal or corrected-to-normal visual acuities. They had no risk factors for noninvasive brain stimulation application, as assessed through safety questionnaires. All participants were naïve to tDCS effects. The use of tDCS in the current study was approved by the *Comissió de Bioètica* of the University of Barcelona. Written informed consent was obtained from each participant before the experiment.

### Go/Nogo-Stop-Signal Task (GNG-SST)

**Figure 1:**
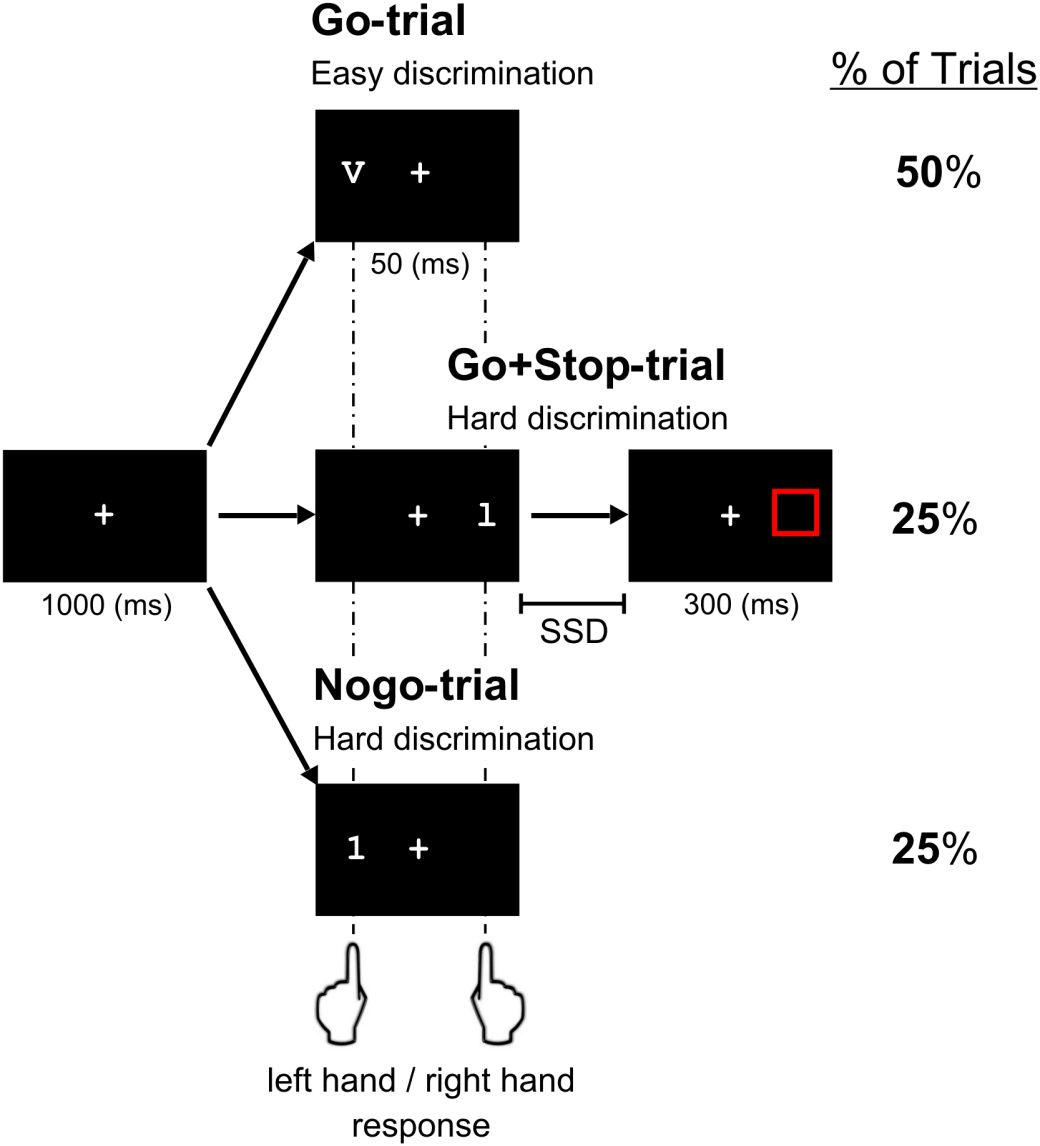
Illustration of the combined GNG-SST designed for the current study. Participants were instructed to respond to letters or numbers in two separated and consecutive blocks with the right or left hand depending on the side of the appearance of the Go-stimuli. In this example, different conditions are shown in the three rows of the Figure for the block “go for letters”, in which participants are asked to respond to the side of appearance of the letters V and l (easy and hard discriminability, respectively). In the top row is presented an example of a Go-trial (letter “V”) for the easy discrimination condition, whereas in the middle row an example of Stop-trial is illustrated for the hard discrimination condition. The Stop-Signal delay (SSD) was adapted (±25 ms) after each Stop-trial by means of a staircase-tracking algorithm. The bottom row corresponds to a Nogo-trial (number “1”) in the hard discrimination condition. The % of Trials (right column) describes the percentage of trials in each condition of a total of 452 trials composing the task.

We implemented a new experimental design that aimed to observe at within-subject level both proactive and reactive inhibitory processes. We adapted a choice-reaction GNG task [31], which allowed incorporating a variant of the SST [21]. In this way, the GNG could preserve the original two variant perceptual complexity conditions, consisting in the manipulation of the difficulty in perceiving the Go and Nogo stimuli, and permitted to further evaluate the role of reactive inhibitory process (i.e., [31]). Thus, two letter-digit pairs in Courier new font served as stimuli (0.8° of visual angle), with one pair being easily discriminable (letter: V and number: 5) and another pair being hard to discriminate (letter: l and number: 1). One stimulus at a time was presented on the left or right side of a central fixation cross, requiring either left or right hand responses with the corresponding index finger (see Figure 1). The two response hands and the two types of discriminable items (easy/hard) were equally frequent and randomly presented within each block of the experiment. The stop-signal was a red frame (0.9° of visual angle) that was presented after a variable delay in the same location of the last Go stimulus, indicating participants to inhibit the Go response in those trials. The delay was adapted to each participant’s behavior by means of a staircase-tracking algorithm [33] as follows: the stop-signal delay (SSD) was set to 250 msec at the beginning of the two blocks and was adjusted separately for the easy and hard discriminability conditions. The SSRT was afterwards calculated first individually for each block and condition and then averaged for each condition. After a successful response inhibition, the SSD was increased by 25 msec, whereas after an unsuccessful inhibition, the SSD was reduced by 25 msec, making the inhibition easier or harder, respectively, in the next stop trial. This dynamic tracking procedure yielded an overall ratio of p(response|stop-signal) of 0.5. Participants were instructed to respond to letters or numbers in two separated and consecutive blocks that were counterbalanced across participants. The Go stimuli were presented for 50 msec, whereas the duration of the stop-signal was always 300 msec. Stimulus onset asynchrony was fixed to 1000 msec. Total number of trials was 432 in each block, for which 50% of the trials corresponded to Go responses, 25% to Nogo responses and 25% to Go+Stop responses. The following constraints were introduced into the task: i) no more than three consecutive stimuli appeared on the same side, ii) two consecutive stop trials never occurred, and iii) the same type of stimuli (either letters or numbers) was not presented more than three consecutive trials in a row.

### Procedure

Each subject participated in two experimental sessions (sham vs. anodal tDCS) counterbalanced across participants and conducted at least one week apart. The experiment began with a practice block that consisted of 64 trials to familiarize the participants with the task. To guarantee that participants began the task aware of the difference between “l” and “1”, the practice block was repeated until the number of commission and omission errors were equal or less than 10. The practice block lasted for ∼4 min, and it was repeated for all participants except for 3. Only 1 subject needed 3 practices blocks to achieve the defined criterion. In the second session, a single practice block was administered, with the only purpose of reminding participants of the task procedure. After every 29 trials, a short break of 5 sec was included to allow participants to rest, and after 144 trials, a 10 sec break was given. Each experiment session lasted for approximately 23 min.

### tDCS

In both experimental sessions the location of the tDCS electrodes was established following the Jacobson et al. [17] study. Thus, in accordance with the 10–20 EEG-system, the anodal electrode was placed on the crossing point between the T4-Fz and F8-Cz positions, whereas the cathodal electrode was placed on the crossing point between the T3-Fz and F7-Cz positions, corresponding to the location rIFC and left IFC on the scalp, respectively.

In the anodal condition, a direct current of 1.5 mA was induced by two square saline-soaked surface sponge electrodes (9 cm^2^; current density 0.16 mA/cm^2^) and delivered by a battery, constant-current stimulator (BrainStim, www.brainstim.it) for 18 min. An automatic on and off ramp of 10 sec was used. In the sham condition, the intensity of the current was the same, but the duration of the stimulation was limited to the duration of the current being ramped on and off (over 20 sec) at the beginning and at the end of the 18 min period. By following this protocol in the sham tDCS session, we ensured that participants felt the same sensations that they felt in the anodal stimulation session.

Importantly, participants were not informed about the different stimulation protocols until the end of the entire experiment, and they could not distinguish between the anodal and the sham tDCS, as assessed by subject responses on a questionnaire completed at the end of each session [34] (nonparametric Wilcoxon rank sum tests all *p*-values>0.08; p = 0.08 corresponded to the value obtained with the rating of fatigue caused by tDCS, with the higher values observed for sham tDCS. Importantly, for the question regarding the influenced tDCS in the performance of the task, we obtained a p-value of 0.32 in the Wilcoxon test).

## Results

For all the analyses the same two factors were introduced in separated repeated measures ANOVAs with two within-subjects factors: tDCS-session (sham vs. anodal) and discriminability (easy vs. hard).

Participants inhibited approximately half of the stop trials in both tDCS-session, indicating a correct implementation of the tracking algorithm [p(response|stop-signal), sham-easy: 49.3±3.6%; sham-hard: 49.1±3.9%; anodal-easy: 47.8±4.0%; anodal-hard: 47.0±3.9%]. Only in the anodal session the p(response|stop-signal) was significantly less than expected (50%) [sham, easy and hard, *p*>0.2; anodal-easy: *t*(21) = −2.6; *p*<0.02; anodal-hard: *t*(21) = −3.7; *p*<0.01]. ANOVA results revealed a main effect of tDCS, denoting that participants inhibited a significantly larger number of trials in the anodal than in the sham tDCS-session [*F*(1,21) = 9.6; *p*<0.01]. A trend towards a significant difference was observed for the discriminability factor [*F*(1,21) = 3.5; *p* = 0.077], which indicated a propensity toward responding to more trials for the hard than for the easy condition.

**Figure 2:**
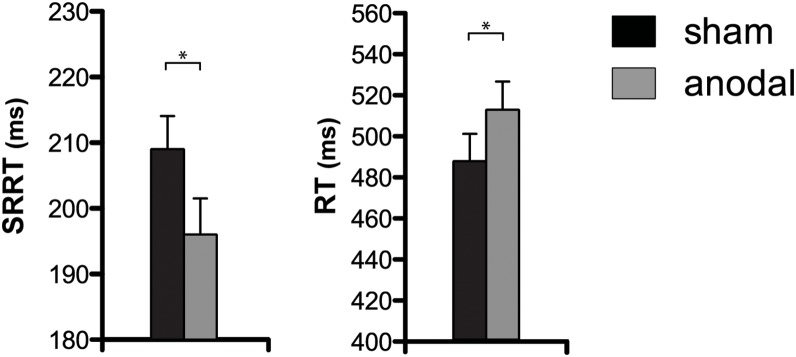
Mean reaction times (±SEM) for Stop-Signal Reaction Time (SSRT), Stop-Signal Delay (SSD) and Go trials for sham and anodal tDCS sessions; the easy and hard discriminability conditions were collapsed. Asterisks represent p-values: *p<0.05; **p<0.01; ***p<0.001; *n.s.* represents statistically non-significance differences (*p*>0.05).

The main results are summarized in Table 1 and Figure 2. The most-used measure for the inhibitory process is the SSRT, which reflects the covert time it takes to suppress a response. Following the integration method, the point at which the stop process finished was estimated as the time corresponding to the *n* ^th^ RT, with *n* equal to the number of RTs in the RT distribution multiplied by the overall p(respond|stop-signal) [35]. The SSRT was then calculated by subtracting the mean SSD from the *n* ^th^ RT separately for each block and condition. ANOVA revealed a significant main effect of tDCS-session [*F*(1,21) = 8.4; *p*<0.01], reflecting a behavioral inhibitory improvement caused by the anodal stimulation. No significant effects were found for the discriminability factor or interaction term (all *p*-values>0.05). However, and for the purpose of further exploring the data, a subsequent post-hoc analysis was conducted which revealed that a significant effect of tDCS-session was observed in the easy-discriminability condition [*t*(21) = 2.6; *p*<0.02]. For the hard-discriminability condition, a trend towards significance was found [*t*(21) = 1.9; *p* = 0.078]. Comparable results were obtained when analyzing the SSRT using the mean method (see Table 1), in which the SSRT is calculated by subtracting the mean SSD from the mean RT. An abundant set of studies has proven the reliability of these two methods.

**Table 1:** Behavioral data.

		Sham	Anodal	*t*-value(d.f. = 21)	*p*-value
		ms (S.D.)	ms (S.D.)		
**GO-RT**	*Easy*	472 (66)	501 (70)	−2.68	<0.02
	*Hard*	502 (60)	524 (61)	−2.05	= 0.05
**SSD**	*Easy*	251 (68)	292 (78)	−3.25	<0.01
	*Hard*	284 (62)	312 (65)	−2.41	<0.03
**SSRT** (integration meth.)	*Easy*	212 (27)	196 (26)	2.56	<0.02
	*Hard*	206 (26)	197 (29)	1.86	= 0.08
**SSRT** (mean meth.)	*Easy*	221 (22)	209 (16)	2.34	<0.03
	*Hard*	219 (21)	212 (25)	1.35	>0.19
		% (S.D.)	% (S.D.)		
**Go-Correct**	*Easy*	98.2 (1.9)	97.7 (2.4)	0.88	>0.3
	*Hard*	90.0 (5.1)	89.9 (6.9)	0.11	>0.9
**False Alarms**	*Easy*	2.6 (3.2)	1.1 (1.5)	2.55	<0.02
	*Hard*	19.4 (14.3)	17.6 (14.4)	0.54	>0.5
**Omissions**	*Easy*	1.8 (1.8)	2.2 (2.4)	−0.91	>0.3
	*Hard*	10.0 (4.9)	10.1 (6.9)	−0.13	>0.8

Values of all different parameters analyzed in the task are shown, separated for easy and hard discrimination conditions and for sham and anodal tDCS sessions. For Go trials, three mean values are presented for easy and hard discrimination conditions: RTs, Percentage of correct responses and omissions. False alarms represent errors in Nogo trials. Mean stop-signal delay (SSD) refers to the average (SSD) in the two blocks (go for letters, go for numbers), computed with different staircases (see Materials and Methods). The Stop-Signal Reaction Time (SSRT) values presented were computed for each subject and condition using both the integration and the mean methods.

The SSRT improvement observed in the anodal tDCS-session was mainly driven by the large increase in the SSD, which was augmented by 30–40 msec under the effects of the anodal tDCS [*F*(1,21) = 8.7; *p*<0.01]. The discriminability factor was also found to modulate the SSD, as revealed by the significant effect [*F*(1,21) = 50.3; *p*<0.001] and a trend, albeit non-significant, towards an interaction of discriminability by tDCS-session [*F*(1,21) = 3.9; *p* = 0.060]. Post-hoc analysis showed a larger increase of the SSD in the easy (41 msec) than in the hard (29 msec) condition caused by anodal tDCS (see Table 1).

When analyzing the RT for Go trials, a strong main effect of discriminability was found [*F*(1,21) = 61.8; *p*<0.001], which indicated that this manipulation worked as expected. Importantly, a significant main effect of tDCS-session was found for Go RT [*F*(1,21) = 6.0; *p*<0.03]. The interaction was not significant [*F*(1,21) = 2.2; *p*>0.1]. The analysis of commission errors revealed only a main effect of discriminability [*F*(1,21) = 50.2; *p*<0.001; tDCS-session: *F*(1,21) = 0.8; *p*>0.3; tDCS-session by discriminability: *F*<0.1], although a significant reduction of commission errors was observed for the easy discriminability condition during the anodal tDCS condition [*t*(21) = 2.5; *p*<0.02]. Finally, the analysis of omitted responses revealed again only a main effect of discriminability [*F*(1,21) = 61.5; *p*<0.001; tDCS-session and tDCS-session by discriminability: *F*<0.2]. Thus, participants did not increase omission rates during the anodal tDCS condition, but a significant reduction of commission errors was observed for the easy discriminability condition, as it could be predicted by the speed-accuracy trade-off, yielding a slight improvement in response accuracy and indicating that tDCS applied over the rIFC could modify both reactive and proactive processes simultaneously.

In order to further testing whether discriminability modulated performance in a considered general inhibitory function, we conducted a Multivariate Analysis of Variance (MANOVA) in which Z-scores of RT and SSRT values were entered into the analysis as dependent variables and discriminability and tDCS-session as fixed factors. The results of this analysis revealed a main effect of tDCS-session [*F*(2,83) = 3.03; *p* = 0.054], while the factor discriminability [*F*(2,83) = 1.86; *p*>0.1] and the interaction of tDCS-session by discriminability [*F*(2,83) = 0.17; *p*>0.8] did not reach statistical significance.

Finally, we conducted a set of Pearson correlation analyses between RT, Commission and Omission and SSRT values with the aim to explore a possible relation between reactive and proactive inhibitory behavior. These analyses were computed separately for easy and hard discrimination condition and considering only the effects of the tDCS (anodal minus sham). The results did not reveal any significant correlation (all *p*-values>0.5), thereby indicating certain level of independency between the two studied inhibitory processes.

## Discussion

This study evaluated the specific role of the rIFC in behavioral inhibition, its relation with signal detection processes, and that effective inhibitory response within the same region by either reactive inhibition or proactive inhibition or both. We assessed this by combining the use of anodal tDCS over rIFG while participants were performing a task that combined, at within subject level, two classical tasks oriented to specifically target inhibitory responses by engaging reactive (i.e., GNG task) and proactive (i.e., SST task) processes. Anodal tDCS slowed down the RT on Go trials, and this result was accompanied by a significant reduction in commission errors when the discriminability of stimuli was easy, although a significant interaction discriminability per tDCS-session did not reach statistical significant levels. These results point out to a speed-accuracy trade-off, thereby indicating a possible increase in task control, which was interpreted as an index of a proactive inhibitory process. Additionally, under tDCS stimulation, the SSRT, a covert index of the inhibitory process, was reduced, demonstrating an improvement in reactive inhibition. Thus, although the activity of the rIFC is directly related in the SST and GNG tasks, the neural mechanisms through which the rIFC plays a role in their execution differs, engaging proactive and reactive inhibitory processes concurrently when needed. Therefore, our data supports a view in which the rIFC is characterized as a key area for behavioral inhibition, and supports the view that the same brain structure could be engaged in a distinct and adaptive manner to inhibit behavioral responses [29], [30].

A variety of tasks has been used for the assessment of response inhibition in a large set of studies. However, none of these studies explored whether two typically differentiated processes like proactive and reactive inhibition can be manipulated concurrently through the influence of the same brain structure, namely the rIFC. In fact, previous studies have noted that such methodological difficulty resided in the high degree of functional similarity of these two processes [36] or postulated that they just co-occur during behavioral inhibition, making it difficult to differentiate one from the other [37]. We resolved this issue by experimentally combining the GNG and SST in a single task –which at the same time triggered the inhibitory processing needed to perform the task by external and internal signals– and by modifying neural excitability of the rIFC by anodal tDCS. The rIFC is recruited in many different task conditions that require sustained attention, and has been proposed as a key cortical area for implementing attentional monitoring and attentional detection rather than inhibition [38], [39]. Taking this idea one step further, the inhibitory function has been proposed to be unnecessary for executive control [36], [40], [41]. However, the rIFC has been functionally and anatomically differentiated into two separate areas, with the ventral posterior part of rIFC being primarily in charge of a global and reactive inhibition process and the dorsal part of IFC being primarily delineated by its role in executive control processes and updating [19], [20].

Although the GNG and SST are the most commonly used tasks for assessing behavioral inhibition, we believe that in the current study they engage different cognitive operations in their execution. In the current GNG task, subjects select the response strategy at the beginning of the block, thereby requiring the subjects to constantly adjust their responding threshold such that a motor response can be withheld before it is initiated [27]. This process is considered to be under proactive control, because task goals are maintained active during the period in which they are required. In the current study we state that a proactive process was engage mainly due to the difficulty of the task that forced the subject for optimizing the response preparation [7]. Our results showed that such phenomenon is reflected as a slower RT and a reduced number of commission errors in the less demanding discriminability condition. In contrast, the SST is specifically efficient in minimizing decision-making because stopping a response implicates withdrawing a motor command already initiated when the stop-signal appears [4], [42].

In terms of cognitive control and under reactive control, goal representations are only retrieved at the time they are needed, giving the advantage of its computational efficiency due to the fact that resources are freed up after the inhibitory command is executed [7]. Crucially, both processes are described as compatible in the DMC framework [7], in terms of the required attentional commitment, because albeit proactive control entails the continuous maintenance of task goals and it forcedly depends on manipulating attentional resource, reactive control is defined as a stimulus driven and transient process that does not make great demands on attentional control [7]. Given that we observed behavioral improvement in both proactive and reactive inhibition processes by experimentally manipulating one single brain region, i.e., the rIFC, we conclude that this brain region may be at least partially responsible for an efficient performance in these two processes.

The recruitment of inhibitory and control mechanisms may explain the extensive activation encountered within the rIFC in most of the fMRI studies using variants of SST that increase decision-making demands and strategy adjustments [39]. In this vein, Verbruggen and Logan [26] in a series of five experiments found that subjects made proactive response-strategy adjustments, increasing response threshold, when they expected the stop-signal to occur. The authors found longer SSRT in selective than in nonselective stopping, i.e., when two different tones were presented but only one was assigned as stop-signal.

In addition, we found that the characteristics of the stimuli did not differently affect the execution of reactive and proactive inhibitory processes, although a small but significant effect was favored in the condition where the difficulty of task was reduced. Hence, in the condition in which the stimulus was easy to discriminate, participants committed less number of errors. Noteworthy, the SSD was significantly larger for the hard than for the easy condition, leaving the possibility that the categorization of the stimulus may be related to the inhibitory response process. Previous neuroimaging studies have investigated the distinct cognitive processes that are involved in stopping, such as response inhibition, attentional capture of the stopping stimulus, and error monitoring [43]. In this vein, Chevrier and colleagues [43] demonstrated that in the Go as well as in the Nogo phase of the SST, both the inhibitory function and performance monitoring interact, especially in unsuccessful stop-trials, activating the regions of the middle PFC associated with response conflict (see also [44]). However, when considering together key measures of proactive and reactive inhibition in the current study, i.e., RT and SSRT values respectively, we did not find that discriminability affected the inhibitory performance.

One important aspect of the present study was the opportunity to employ tDCS to modulate a cognitive function. The mechanism underlying the neuromodulatory effects induced by tDCS are well established. Several studies using animal models have suggested that tDCS can promote effective excitation or inhibition of neurons in a polarity-specific manner [45], [46]. Accordingly, it has been shown that anodal depolarization increases cortex excitability [47], [48]. In a recent tDCS study, Jacobson et al. [17] employed a SST and tested different sets of stimulation and demonstrated that anodal tDCS applied unilaterally over the rIFC yielded the largest improvements in reactive inhibition, as reflected in a reduction of the SSRT. The most significant difference between the results from Jacobson and colleagues [17] and the current results is that we observed a significant slowing of RTs for Go trials in the anodal relative to the sham session. This difference can only be explained by an enhanced proactive control following anodal tDCS of rIFC.

Some results of stop-signal studies have shown that subjects trade speed in Go trials for success in stopping after stop-signal trials [49], [50] and that subjects trade speed for accuracy in stopping in blocks where the stop-signal is presented (e.g., [35]). However, perhaps the most robust effect that demonstrates proactive behavioral adjustments is found in studies in which the proportion of stop-signals is manipulated, establishing that slower RTs are found when the proportion of stop-signals increases [25], [26] and suggesting an increase in proactive inhibition. Remarkably, in the current study, slower RTs were due to application of anodal current over the rIFC, supporting a direct link between this cortical region and proactive inhibition.

## Conclusions

Reactive and proactive inhibitory processes are considered to be an integral part of cognitive control [2] of executive functions. The major contribution of the current study is to show the simultaneous enhancement and interrelation of these two traditionally separated cognitive mechanisms when modulating the neural activity of rIFC. Altered control in response inhibition has been related to several dysfunctions, most notably to attention-deficit hyperactivity disorder [51]. In addition, recent clinical studies have proposed the rIFC as a candidate for altered cognitive control in diseases like schizophrenia [52] or obsessive compulsive behavior [53]. Given that we found improvements in proactive and reactive processes after anodal tDCS of rIFC, a key question in future studies would be to evaluate if anodal tDCS would be a useful tool to remediate inhibition deficits associated with the afore-mentioned disorders (but see [18]). Such work would be important for furthering the knowledge of these processes and for developing innovative clinical treatments.

## Supporting Information

Data S1 Data summary. Average data for all subjects on the different measurements obtained in the task are provided, as well as data used for computing the SSRT with the integration method (percentage of correct inhibition, distribution of the RT and the SSD) and mean method (SSD and mean RT).
(XLSX)
